# Cannabidiol reshapes the gut microbiome to promote endurance exercise in mice

**DOI:** 10.1038/s12276-025-01404-5

**Published:** 2025-02-18

**Authors:** Si Chen, Yu-Bin Lee, Mi-Young Song, Changjin Lim, Hwangeui Cho, Hyun Joo Shim, Jong-Suk Kim, Byung-Hyun Park, Jeon-Kyung Kim, Eun Ju Bae

**Affiliations:** 1 Department of Biochemistry and Molecular Biology, Jeonbuk National University Medical School, Jeonju, Republic of Korea; 2 School of Pharmacy and Institute of New Drug Development, Jeonbuk National University, Jeonju, Republic of Korea; 3 Graduate School of Medical Science and Engineering, Korea Advanced Institute of Science and Technology, Daejon, Republic of Korea

**Keywords:** Molecular biology, Biological therapy

## Abstract

Cannabidiol (CBD), a nonpsychoactive compound from *Cannabis*, has various bioactive functions in humans and animals. Evidence suggests that CBD promotes muscle injury recovery in athletes, but whether and how CBD improves endurance performance remains unclear. Here we investigated the effects of CBD treatment on exercise performance in mice and assessed whether this effect involves the gut microbiome. CBD administration significantly increased treadmill running performance in mice, accompanied by an increase in oxidative myofiber composition. CBD also increased mitochondrial biogenesis and the expression of associated genes such as PGC-1α, phosphorylated CREB and AMPK in muscle tissue. Interestingly, CBD altered the composition of the gut microbiome, and antibiotic treatment reduced the muscle endurance-enhancing effects of CBD and mitochondrial biogenesis. We isolated *Bifidobacterium animalis*, a microbe increased by CBD administration, and named it KBP-1. Treatment with *B. animalis* KBP-1 in mice resulted in improved running performance. Whole-genome analysis revealed that *B. animalis* KBP-1 presented high expression of genes involved in branched-chain amino acid biosynthesis, expression of branched-chain amino acid release pumps and metabolism of lactic acid. In summary, our study identified CBD and *B. animalis* KBP-1 as potential endurance exercise-promoting agents.

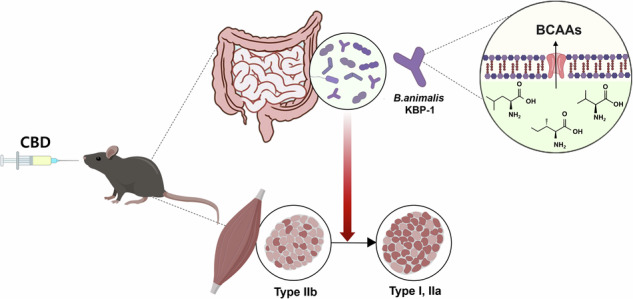

## CBD enhances endurance performance through gut microbiome changes

This study explores how cannabidiol (CBD), a compound from the *Cannabis sativa* plant, affects exercise performance and muscle function. The researchers wanted to see if CBD could improve endurance by changing gut bacteria. They found that CBD improved endurance and increased the presence of certain gut bacteria, including *Bifidobacterium animalis*, that may help muscles use energy more efficiently. The study involved treating mice with either CBD or *B. animalis* for 4 weeks and measuring their running ability on a treadmill. The researchers examined changes in muscle fibers and gut bacteria composition. They discovered that CBD and *B. animalis* increased oxidative muscle fibers, which benefit endurance performance. The results suggest that CBD enhances exercise performance by promoting beneficial gut bacteria and improving muscle energy use. The researchers conclude that both CBD and *B. animalis* could be used to boost endurance.

## Introduction

Understanding the fitness and adaptability of skeletal muscle is fundamental for evaluating exercise capacity^1^. Skeletal muscle comprises type I and type II fibers with distinct myosin heavy chain (MyHC) isoforms that determine contraction rates, with slow-twitch fibers abundant in the soleus (type I) and fast-twitch fibers in the extensor digitorum longus (type II)^2,3^. However, this fiber composition can dynamically adapt to various physiological stimuli, such as endurance exercise, which favors slow-twitch oxidative fiber predominance^4^. Endurance training activates mitochondrial biogenesis signaling pathways to meet metabolic demands, with several key molecules coordinating these complex cellular responses. Notably, AMP-activated protein kinase (AMPK), cAMP response element-binding protein (CREB) and PPARγ coactivator-1α (PGC-1α) have been extensively studied as promising targets for enhancing exercise performance through mitochondrial biogenesis.

The *Cannabis sativa* plant has been used for medicinal and recreational purposes for thousands of years. It contains more than 100 identified phytocannabinoids, primarily Δ9-tetrahydrocannabinol (THC) and cannabidiol (CBD). While THC is the main psychoactive component, CBD is nonpsychoactive and has been investigated for its numerous pharmacological effects, including antipsychotic, antiepileptic, neuroprotective, antidiabetic and anti-inflammatory properties^5^. Currently, Epidiolex is the only U.S. Food and Drug Administration (FDA)-approved CBD prescription specifically for seizures in certain patients with genetic epilepsy. Recently, CBD was removed from the World Anti-Doping Agency’s ‘Prohibited List’^6^, further encouraging its potential applications.

Previous studies have shown that CBD influences skeletal muscle function under various experimental conditions. In myotubes, CBD affects the transcription of a wide variety of genes and protects against oxidative stress^7,8^ but does not influence anabolic or inflammatory signaling^9^. In in vivo studies, CBD has improved the muscular lipid profile in mice and rats fed a high-fat diet^10,11^, enhanced skeletal muscle regeneration after resistance training^12^ and increased muscle strength in Duchenne muscular dystrophy model mice^13^. In humans, however, the effect of CBD consumption on skeletal muscle performance remains controversial^14^. One study revealed that an acute dose of CBD (60 mg) reduced the serum levels of creatine kinase and myoglobin while enhancing one-repetition maximum back squat performance after 72 h. However, two recent clinical trials in healthy adults revealed that neither an 8-week regimen of 50 mg of CBD nor an acute dose of 300 mg of CBD improved aerobic or anaerobic fitness. Thus, it remains to be determined whether and how chronic oral administration of CBD may affect endurance exercise performance. Meanwhile, research on the impact of THC on skeletal muscle function is limited, with most studies concentrating on its antinociceptive effects as a modulator of the endocannabinoid system. THC is an active ingredient in Sativex (nabiximols), a 1:1 THC–CBD mixture that is used to relieve muscle stiffness and spasms in multiple sclerosis.

Exercise performance is closely linked to the gut microbiota^15–19^. Certain bacterial communities enhance endurance and reduce inflammation, whereas exercise itself influences the composition of the gut microbiota. For example, administering *Bacteroides uniformis* to long-distance runners may increase endurance by promoting hepatic glucose production^18^. In addition, increased levels of *Veillonella atypica* in the gut of marathon runners produce propionate from the lactic acid generated during exercise, extending the duration of endurance exercise^19^.

CBD has been shown to alter the gut microbiota, with associated improvements in cognitive function^20^, metabolic syndromes^21^ and autoimmune encephalomyelitis^22^. Therefore, this study investigated whether CBD could enhance exercise performance by modulating the composition of the gut microbiota. Our results indicate that CBD treatment improved exercise performance and mitochondrial function in skeletal muscle in mice, which was mediated by changes in the gut microbiota. We identified a key bacterial responder, *Bifidobacterium animalis*, termed KBP-1, highlighting its potential as an option for promoting endurance exercise.

## Methods

### Animal studies

Male C57BL/6J mice aged 20 weeks were purchased from Samtako Bio Korea (Osan, Korea). The mice were kept under a 12-h light‒dark schedule with food and water available ad libitum at a temperature of 22 °C (±1 °C) and a humidity level of 50% (±10%). The mice were allowed to adjust for 1 week before the experiment began. Body fat and lean body mass were measured with a Minispec mq 7.5 NMR system from Bruker Optics (Germany).

First, we evaluated whether the oral administration of CBD, synthesized in-house following a previously published method^23^, could alter treadmill running performance in mice. CBD, which was stored at −20 °C and protected from light, was freshly dissolved in corn oil (C8267, Sigma-Aldrich) before use and was administered orally at a dosage of 30 mg/kg once daily at 9:00 for 4 weeks. The CBD dosage was determined on the basis of recent research^23^, considering its effective concentration range (nM to μM)^24^ and its poor oral absorption^25,26^.

In the second cohort of mice, we evaluated how antibiotic treatment influences the effect of CBD on treadmill running performance. To achieve this goal, we selected an antibiotic that specifically targets strains increased by CBD administration while minimizing disruptions to the rest of the microbiota on the basis of a comparison of different antibiotic classes. The antibiotic dosage for mice was calculated using human-equivalent doses adjusted for mouse body surface area^27^. Specifically, we used doxycycline (D9891) at 40 mg/kg^28^, clarithromycin (PHR1038) at 100 mg/kg^29^, vancomycin (V2002) at 400 mg/kg (ref. ^30^), metronidazole (M3761, Sigma-Aldrich) at 400 mg/kg^31^ and cefaclor (C3478, Tokyo Chemical Industry) at 150 mg/kg^32^ and identified doxycycline as the most suitable choice. The mice were treated with doxycycline (40 mg/kg) dissolved in sterile saline, either alone or in combination with CBD, while they were subjected to treadmill testing.

In the third cohort of mice, microbes (*Faecalibaculum rodentium* or *Bifidobacterium animalis*) were administered orally to the mice at a dose of 1 × 10^8^ CFU/mouse diluted in sterile saline once daily at 9:00 for 4 weeks. The effects on treadmill running performance were compared with those of CBD. The selected dose for these microbes was based on previous studies and falls within the lower range of reported doses^33,34^.

All experimental procedures were approved by the Institutional Animal Care and Use Committee of Jeonbuk National University (permit no. 2023-153).

### Treadmill exercise assessment

The treadmill running assessment was performed as described in our previous studies with slight modifications^35,36^. The mice were acclimated to a single lane of a 16-lane treadmill (Jeung Do Bio & Plant, Korea) by running for 30 min at 10 m/min daily for 1 week. They were then subjected to a moderate-intensity treadmill running test for 4 weeks, with CBD or a single bacterium administered at least 1 h before training. The test began at a speed of 10 m/min for 10 min, with an increase of 2 m/min every 10 min, reaching a maximum of 16 m/min until exhaustion. Exhaustion was defined as the inability to resume running despite mild stimulation with a wooden cane. The mice ran at the same time during the day each day. Running time and distance were measured during the final 2 weeks of the 4-week study.

### Indirect calorimetry

Each mouse was placed in a separate Oxymax/CLAMS metabolic cage system (Columbus Instruments, USA). They underwent a 24-h acclimatization period within the metabolic cages to minimize stress. Constant surveillance for 72 h in a controlled environment was maintained at a temperature of 22 °C with a 12-h light‒dark cycle (19:00 to 7:00). The Oxymax system recorded the respiratory exchange ratio (RER; VCO_2_/VO_2_) and collected the last 24 h of data for analysis.

### Biochemical analysis

Accu-Chek (Roche) was used to measure blood glucose levels. A CareSens Dual monitoring system (i-SENS) was utilized to measure blood β-hydroxybutyrate levels. Blood lactate levels were determined via the Lactate Pro2 (ARKRAY).

### Histology

Gastrocnemius (GAS) muscle tissues were embedded in liquid-nitrogen-cooled isopentane. A cryomicrotome (Thermo Fisher Scientific) was used to cut the samples at 10 μm, and the muscle sections were placed in a cold container maintained at cryostat temperature. To perform succinate dehydrogenase (SDH) staining, the samples were placed in 0.2 M sodium phosphate buffer solution (pH 7.6) with 50 mM sodium succinate and 0.6 mM nitro blue tetrazolium (Sigma-Aldrich) and then incubated at room temperature for 30 min. Then, the slides were washed with distilled water three times and mounted with aqueous mounting medium (H-5501, Vector Laboratories). Immunofluorescence staining of different MyHC isoforms involved blocking in a 5% goat serum solution followed by incubation with primary antibodies against MyHC1 (BA-F8), MyHC2a (SC-71) and MyHC2b (BF-F3) (all obtained from DSHB) at 4 °C overnight. After the slides were washed three times with phosphate buffer solution, they were incubated with Alexa Fluor 488-conjugated goat anti-mouse IgG1 (A21121), Alexa Fluor 350-conjugated goat anti-mouse IgG2b (A21140) and Alexa Fluor 594-conjugated goat anti-mouse IgM (A21044) secondary antibodies (all from Thermo Fisher Scientific) at 37 °C for 1 h. Images were acquired using a Leica DM750 microscope (Leica). The different types of fiber were counted manually. Image analysis was conducted using iSolution DT 36 software (Carl Zeiss).

### Transmission electron microscopy

GAS muscle tissues were initially fixed in a solution of 2% paraformaldehyde and 2% glutaraldehyde in 50 mM sodium cacodylate buffer (pH 7.2) at 4 °C overnight. The samples were subsequently fixed with 1% osmium tetroxide in 50 mM sodium cacodylate buffer at 4 °C for 1.5 h. The muscle tissues were then stained with 0.5% uranyl acetate overnight at 4 °C and subsequently dehydrated in a graded ethanol series, with each step lasting 10 min at room temperature. The samples were infiltrated with a mixture of propylene oxide and Epon 812 resin (EMS) for viewing and imaging under a Hitachi Bio-TEM system at the Jeonbuk National University Electron Microscopy facility.

### Ex vivo muscle isometric force and fatigue measurement

Freshly excised GAS muscle tissue was immersed in an aerated bath containing saturated Krebs-Ringer solution. The GAS muscle tendons were fastened with 6–0 nylon sutures to stainless-steel hooks, and the muscle was positioned vertically between a force transducer and an adjustable hook (Model 159901, Radnoti). The GAS muscle was placed in an organ bath and continuously exposed to Krebs-Ringer solution at room temperature via platinum electrodes. After equilibration, the muscle was subjected to various stimulation frequencies (10–200 Hz with 2-ms pulses, 100 V for 500 ms, with a recovery interval of 1 min) to measure tetanic force. Fatigue levels were evaluated through multiple stimulations at a rate of 1 Hz and 100 V over a period of 7 min. LabChart Pro software (Version 8, ADInstruments, USA) was utilized for data analysis.

### Mitochondrial respiration

C2C12 cells were acquired from the American Type Culture Collection (ATCC). C2C12 myoblasts were plated in XF24 plates at a density of 20,000 cells per well for Seahorse analysis (XF96, Agilent Technologies). On the sixth day of differentiation, we treated C2C12 myotubes with either vehicle (VEH; dimethyl sulfoxide) or CBD (2 μM). One hour before the assay, the medium was replaced with DMEM containing 5 mM glucose and 1 mM pyruvate. The oxygen consumption rate was measured via the Seahorse XF Cell Mito Stress Test Kit (Agilent Technologies) after three injections: 1 μM oligomycin, 0.5 μM FCCP and 1 μM rotenone/antimycin A.

### Western blotting

Tissue protein extraction reagent was used to homogenize the GAS muscle samples. Protein extracts from muscles were separated via 10% SDS‒PAGE and then transferred onto polyvinylidene difluoride membranes. Blots were treated with 5% skim milk, washed three times and then probed with primary antibodies against phospho-PKA substrate (9624), CREB (9197), p-CREB (9198), p-AMPKα (50081), AMPKα (5831, Cell Signaling Technology), mtTFA (sc-23588), Drp1 (sc-271583), Fis1 (sc-376447, Santa Cruz Biochemicals), Mfn1 (ab57602), T-OxPhos (ab110413, Abcam), PGC-1α (AB-3242, Millipore), OPA1 (612606, BD Biosciences) or HSP90 (ADI-SPA-836-F, Enzo Life Sciences). Immunoreactive bands were detected with a Las-4000 imager (GE Healthcare Life Sciences).

### RNA isolation and real-time quantitative RT–PCR

Total RNA was extracted from frozen GAS muscle tissue via an RNA kit (Invitrogen). First-strand cDNA was produced with the random hexamer primer provided in the first-strand cDNA synthesis kit (K1672, Thermo Fisher Scientific). Specific primers were created via qPrimerDepot (http://mouseprimerdepot.nci.nih.gov) (Supplementary Table 1). PCR was performed in a final volume of 10 µl with 10 ng of reverse-transcribed total RNA, 200 nM of both forward and reverse primers, and a PCR master mixture. An ABI Prism 7900HT Sequence Detection System (Applied Biosystems) was used for quantitative PCR (qPCR) analysis in 384-well plates.

Mitochondrial DNA (mtDNA) content analysis required the extraction of total DNA via a genomic DNA (gDNA) purification kit (Qiagen). The quantity of mtDNA was measured through qPCR using primers targeting the mitochondrial genes Mtco1 and Mtco2 and normalized to the nuclear-encoded gene cyclophilin A.

### 16S rDNA amplicon sequencing

The collected stool samples were preserved in DNA/RNA Shield solution (Zymo Research) and stored at −80 °C until DNA preparation. gDNA was extracted from the stool samples via the QIAamp DNA Microbiome Kit (Qiagen) according to the manufacturer’s instructions. The bacterial gDNA samples were subjected to amplicon sequencing, targeting the V3–4 region of the 16S rDNA gene using the MiSeq platform (Illumina) or the V4 region using the iSeq100 platform (Illumina), generating Fastq files. Quality control and analysis of the data obtained from the MiSeq platform were performed using the DADA2 package (version 1.18.0) in R (version 4.0.3), which corrects sequencing errors and identifies amplicon sequence variants (ASVs) by referencing the National Center for Biotechnology Information (NCBI) 16S Microbial Database. For the sequencing data generated by the iSeq platform, quality control and analysis were performed using the Quantitative Insights Into Microbial Ecology (QIIME2) pipeline, and ASVs were identified via the SILVA database (version 138). The relative abundance, α-diversity (Shannon index, ASVs, and ACE index), and β-diversity (unweighted UniFrac) were analyzed via QIIME2 and visualized via the R software package for relative abundance, and a Plotly Make Chart (https://chart-studio.plotly.com/create/#) was generated for β-diversity. The sequencing reads have been deposited in the NCBI Short Read Archive under accession numbers PRJNA1106047 (https://www.ncbi.nlm.nih.gov/bioproject/?term=PRJNA1106047) and PRJNA1106045 (https://www.ncbi.nlm.nih.gov/bioproject/?term=PRJNA1106045).

### Single bacteria isolation

The feces from the mice treated with CBD were immediately collected and diluted in Gifu Anaerobic Medium broth (Nissui Pharmaceutical, Japan). The samples were subsequently smeared onto blood‒liver agar plates (Nissui Pharma) at a dilution of 1:100,000. The blood‒liver agar plates were cultured under anaerobic conditions in an anaerobic chamber (Mitsubishi Gas Chemical Company, Japan) along with GasPak EZ anaerobic pouches (Becton, Dickinson and Company, USA) to create an anaerobic environment at 37 °C. After cultivation, sequence analysis for identification of the isolated colonies was performed via high-throughput DNA analysis (Bionics, Korea) by the Sanger method using the primers 518 F (5′-CCA GCA GCC GCG GTA ATA CG-3′) and 800 R (5′-TAC CAG GG TAT CTA ATC C-3′) of the 16S rDNA region of the bacteria from which the genes were extracted.

### Whole-genome sequencing

The sequencing libraries were prepared according to the manufacturer’s guidelines for 20-kb template preparation via the BluePippin Size-Selection System and PacBio DNA Template Prep Kit 1.0. Sequencing analysis was performed at CJ Bioscience. Sequencing data were produced via the HGAP2 protocol in PacBio SMRT Analysis 2.3.0 (Pacific Biosciences), and Circlator version 1.4.0 (Sanger Institute) was used for the concatenation of contigs (Sanger Institute). The KBP-1 genome sequence, consisting of 2 contigs, was acquired via the PacBio Sequel platform. The gene-finding and functional annotation pipeline of whole-genome assemblies was used in the EzBioCloud genome database. Protein-coding sequences (CDSs) were predicted via Prodigal 2.6.2^37^. Genes coding for tRNAs were searched using tRNAscan-SE 1.3.1^38^, the rRNAs and other noncoding RNAs were searched via a covariance model search with the Rfam 12.0 database^39^, and CRISPRs were detected via PilerCR 1.06^40^ and CRT 1.2^41^. The CDSs were classified on the basis of their roles concerning orthologous groups (EggNOG 4.5; http://eggnogdb.embl.de)^42^. For more functional annotation, the predicted CDSs were compared with the SwissProt^43^, Kyoto Encyclopedia of Genes and Genomes^44^ and System for Exchange of Excise Data^45^ databases via the UBLAST program^46^.

### Analysis of SCFAs via LC‒MS

Blood and fecal short-chain fatty acid (SCFA) quantification was performed via 3-nitrophenylhydrazine derivatization followed by liquid chromatography‒mass spectrometry (LC‒MS). Standard solutions or samples were mixed with 3-nitrophenylhydrazine, 1-ethyl-3-(3-dimethylaminopropyl)carbodiimide and pyridine solution and then reacted at 40 °C. The reaction mixture was diluted with the starting mobile phase for LC‒MS/MS analysis. Separation was performed using a reverse-phase C18 column with gradient elution, using 0.1% formic acid in water and acetonitrile as the mobile phase. Mass spectrometric detection was conducted in negative-ion mode with a triple quadrupole mass spectrometer, and multiple reaction monitoring and electrospray ionization were used.

### Statistical analysis

The data are presented as the mean ± standard error of the mean. All values indicated by dots in the figures refer to biological replicates. The significance of differences was determined using two-tailed Student’s unpaired *t*-tests. *P* < 0.05 was considered significant. GraphPad Prism 9.4 software was used to conduct the statistical analyses.

## Results

### CBD enhances exercise endurance and promotes muscle glycolytic-to-oxidative fiber type transition

**Fig. 1:**
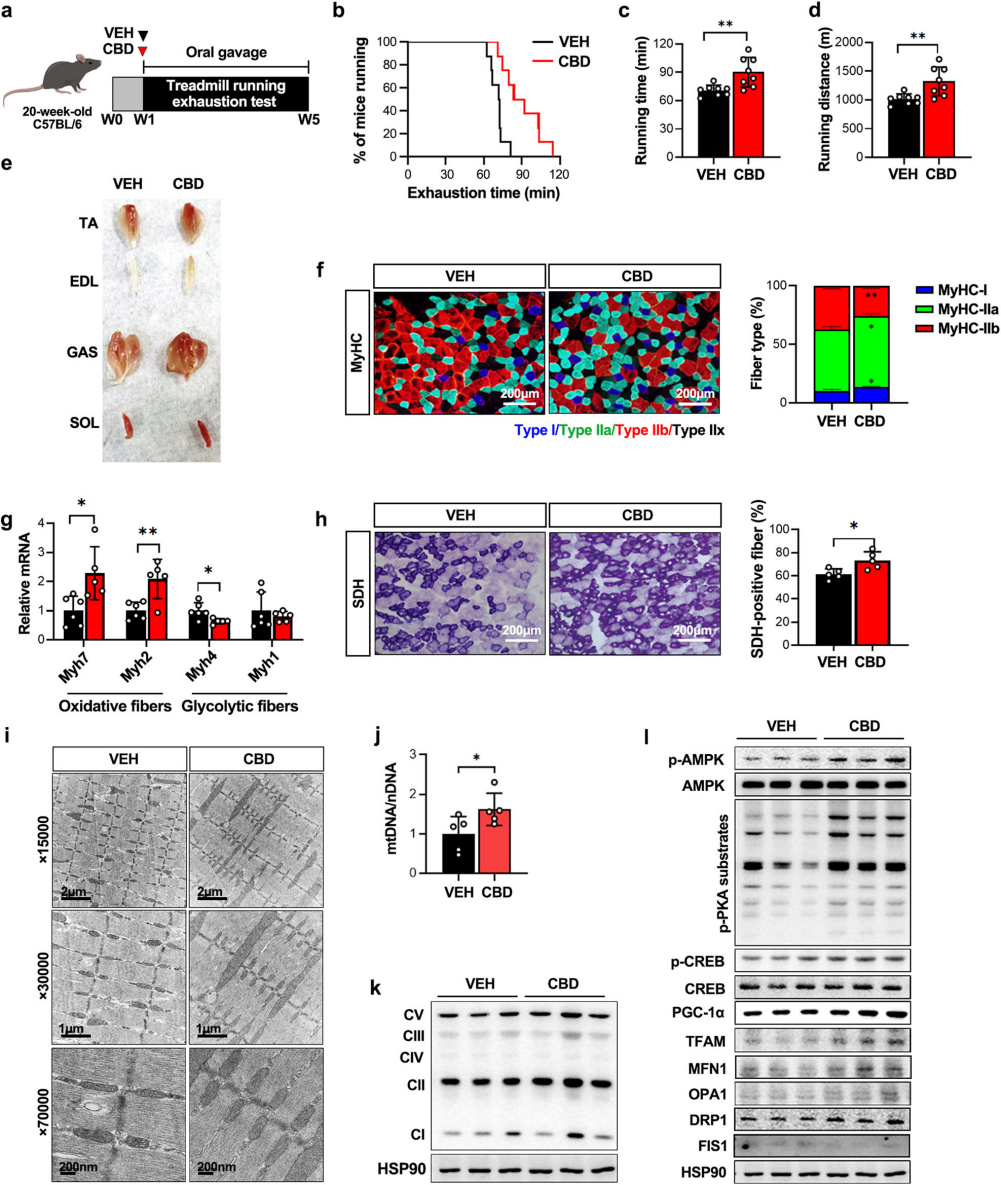
CBD administration increases endurance exercise performance and mitochondrial biogenesis in mice. **a** Schematic of a study measuring CBD-mediated exercise performance enhancement. **b** Running population plotted against time to exhaustion (*n* = 8). **c**, **d** Average running time (**c**) and distance until exhaustion (**d**) (*n* = 8). **e** Representative photographs showing skeletal muscles from VEH- and CBD-treated mice. **f** Microscopy images of GAS muscles immunostained with anti-MyHC antibodies (*n* = 5). **g** qPCR was utilized to examine the mRNA expression levels of known markers for type I and type II muscle fibers (*n* = 5–6). **h** Quantification of SDH-positive fibers in GAS muscles via SDH staining (*n* = 5). **i** Transmission electron micrographs of GAS muscles in mice treated with CBD or VEH. **j** qPCR was used to quantify mtDNA levels compared with nuclear DNA (nDNA) levels (*n* = 5). **k** Western blot analysis of the expression of mitochondrial biogenesis genes in GAS muscles (*n* = 6). **l** Western blot analysis of the expression of OxPhos subunits in GAS muscles (*n* = 6). *P < 0.05, **P < 0.01. TA, tibialis anterior; EDL, extensor digitorum longus; GAS, gastrocnemius; SOL, soleus.

We first investigated whether CBD treatment improves exercise performance in mice. Twenty-week-old C57BL/6 mice were acclimated to a treadmill, running for 30 min daily at 10 m/min for 1 week, and then divided into two groups: those treated with corn oil (VEH) and those given CBD orally at 30 mg/kg/day for 4 weeks (Fig. 1a). During treatment, the mice were subjected to a daily involuntary treadmill running test. The results revealed increased endurance in the CBD-treated group, as indicated by increased running distance and extended time to exhaustion (Fig. 1b–d), with no changes in body weight or food intake (Supplementary Fig. 1a, b). After euthanasia, hind limb muscles appeared more red in CBD-treated mice (Fig. 1e), indicating a shift toward oxidative muscle fibers. To examine fiber composition, we analyzed the fiber type and cross-sectional area of hind limb muscles. Immunofluorescence staining of MyHC isoforms revealed that type I and II oxidative fibers (MyHC-I and MyHC-IIa) were increased whereas type II glycolytic fibers (MyHC-IIb) were decreased in the GAS muscle following CBD treatment (Fig. 1f). Similarly, qPCR revealed increased mRNA expression of *Myh7* and *Myh2* (encoding MyHC-I and MyHC-IIa, respectively), while *Myh4* (MyHC-IIb) expression was decreased, and *Myh1* (MyHC-IIx) expression remained unchanged (Fig. 1g). SDH staining revealed that more than 70% of muscle fibers were SDH positive in the CBD group, compared with 60% in the VEH group (Fig. 1h). Indirect calorimetry analysis revealed a lower RER (VCO_2_/VO_2_) in the CBD group, indicating a shift from glucose to fatty acid oxidation (Supplementary Fig. 1c). Furthermore, ex vivo muscle function analysis revealed that GAS muscles from CBD-treated mice exhibited stronger tetanic contraction and were more resistant to fatigue (Supplementary Fig. 1d–f).

### CBD increases mitochondrial content and oxidative capacity and activates the PKA–CREB–PGC-1α pathway

Because an increase in the oxidative capacity of muscle fibers is associated with mitochondrial biogenesis and function^47^, we measured changes in mitochondrial content and related gene expression in GAS muscles from CBD-treated mice. Electron microscopy revealed a greater prevalence of fused intermyofibrillar mitochondria in the CBD group than in the control group, suggesting improved oxidative phosphorylation (OxPhos) capacity (Fig. 1i). The administration of CBD significantly increased the mtDNA content, as assessed by the mitochondrial genome-to-nuclear genome ratio (mtDNA/nDNA) (Fig. 1j). Western blot analysis revealed increased OxPhos protein levels in complexes I–III and V (ATP synthase) in CBD-treated muscles (Fig. 1k and Supplementary Fig. 1i), which was supported by the qPCR results of genes related to mitochondrial biogenesis and OxPhos (Supplementary Fig. 1j). Seahorse XF Mito stress tests in C2C12 myoblasts revealed increased maximal respiration after CBD treatment (Supplementary Fig. 1g). These findings suggest that CBD enhances muscle oxidative capacity through mitochondrial biogenesis and dynamic alterations.

Finally, we performed western blot analysis to measure the key signaling pathways and transcription factors involved in mitochondrial biogenesis and the maintenance of muscle integrity. The results confirmed that CBD activated AMPK and PKA, as indicated by increased p-AMPK and p-PKA substrate levels, along with elevated downstream CREB phosphorylation and PGC-1α levels (Fig. 1l and Supplementary Fig. 1h).

### CBD changes the composition of the gut microbiome, and antibiotic treatment inhibits its exercise performance-enhancing effects

**Fig. 2:**
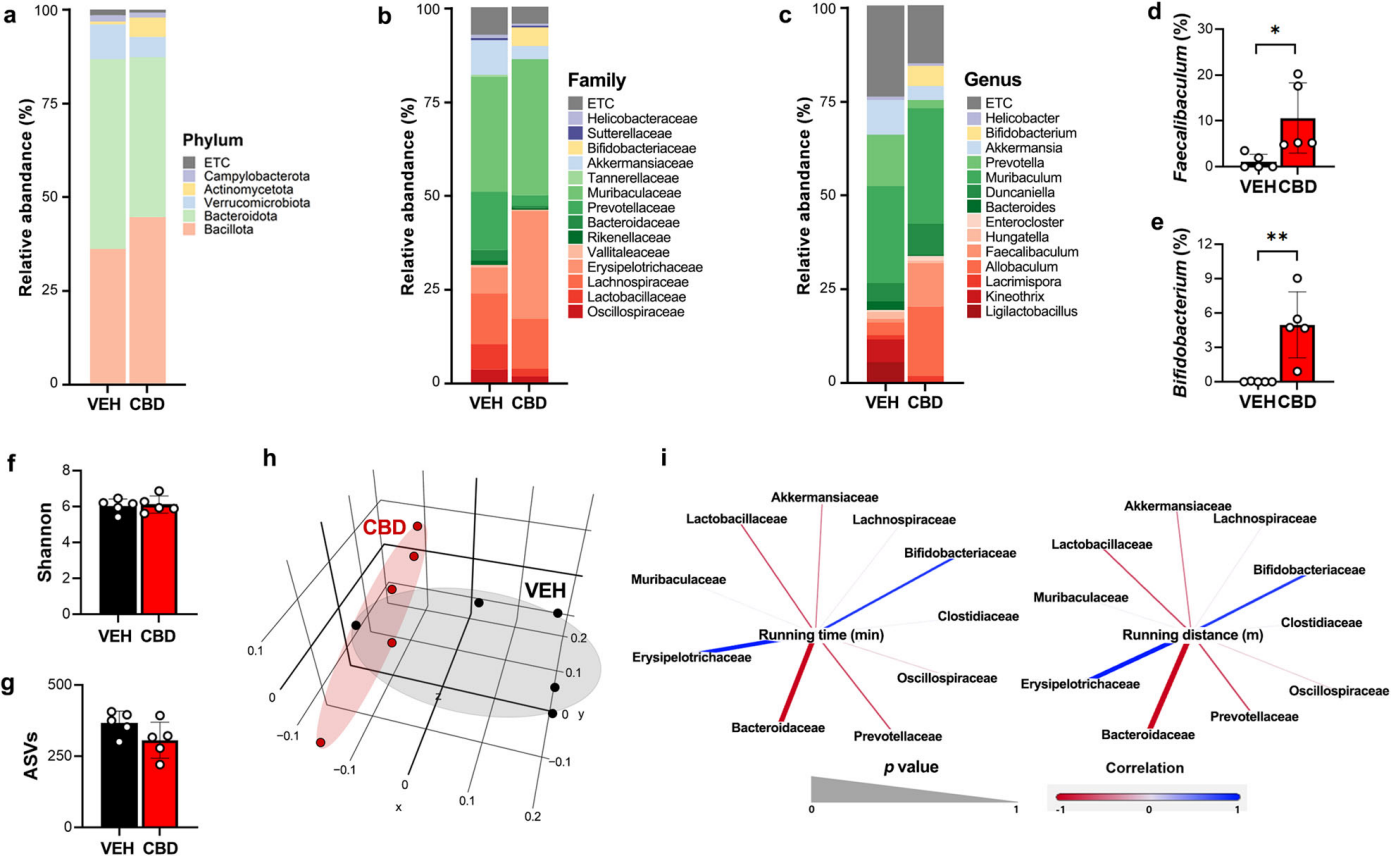
CBD administration changes the gut microbiome. **a**–**c** The relative abundance of bacteria, as analyzed through 16S rDNA amplicon sequencing, at the phylum (**a**) family (**b**) and genus (**c**) levels (*n* = 5). **d**, **e** Comparison of the ratios of *Faecalibaculum* (**d**) and *Bifidobacterium* (**e**) in the VEH and CBD treatment groups (*n* = 5). **f**, **g** The α-diversity of the VEH and CBD treatment groups was assessed using the Shannon index (**f**) and ASVs (**g**) (*n* = 5). **h** β-Diversity analysis was performed with unweighted UniFrac metrics (*n* = 5). **i** The correlation analysis between running time or distance and the relative abundance at the family level was generated via Gephi (version 0.9.7) (*n* = 5). *P < 0.05, **P < 0.01.

Given the established association between the gut microbiome and athletic performance^15–17^, we investigated whether CBD-induced changes in the microbiome might contribute to improved endurance. 16S rRNA sequencing of fecal samples revealed significant changes in microbiome composition with CBD treatment. At the phylum level, the CBD treatment increased the abundance of Bacillota and Actinomycetota (Fig. 2a), whereas at the family level, there was a significant increase in the proportion of Erysipeltrichaceae and Bifidobacteriaceae and a substantial decrease in the proportion of Oscillospiraceae and Pervotella (Fig. 2b). At the genus level, there was a significant increase in the proportion of *Allobaculum* and *Faecalibaculum* (Erysipeltrichaceae family) and *Bifidobacterium* (Bifidobacteriaceae family) (Fig. 2c–e). While α-diversity indices (Shannon indices and ASVs) did not significantly change (Fig. 2f, g), principal coordinate analysis confirmed a significant separation between groups (*P* = 0.03) (Fig. 2h), indicating a distinct microbiome shift. We focused on microbes from the Erysipelotrichaceae and Bifidobacteriaceae families, which were positively correlated with improved muscular endurance due to CBD administration (Fig. 2i).

To investigate causality, we performed an intervention study with antibiotics (ABX) alongside CBD. As the gut microbiome can cause weight change and directly affect exercise performance^48^, we compared different classes of ABX. Among various ABX, doxycycline was selected for minimal microbiome disruption while targeting Erysipelotrichaceae and Bifidobacteriaceae (Supplementary Fig. 2).

**Fig. 3:**
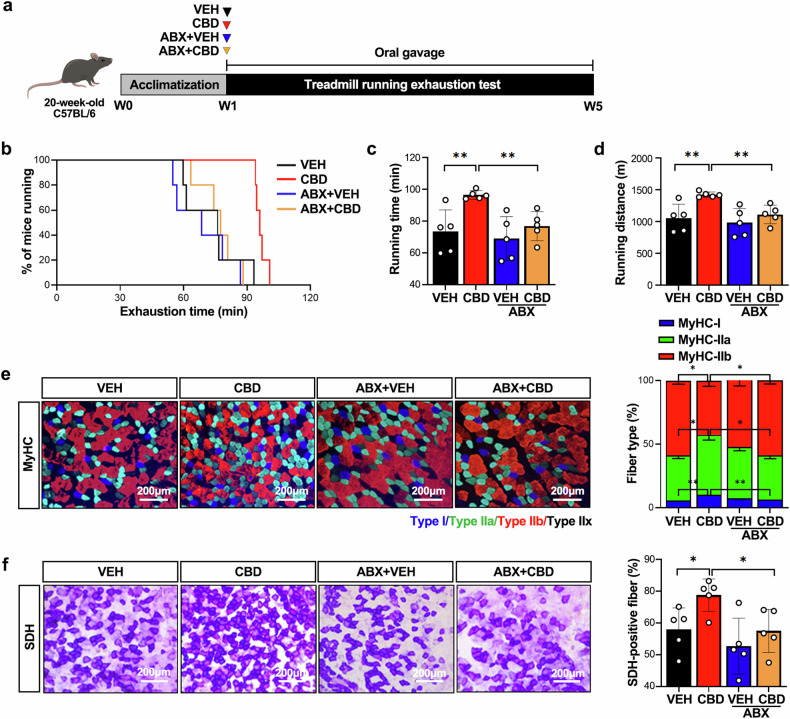
The administration of ABX offsets the effect of CBD on increased exercise performance. **a** Experimental design for assessing the effects of CBD with and without ABX on exercise performance. **b** Running population plotted against time to exhaustion (*n* = 5). **c**, **d** Average running time (**c**) and distance until exhaustion (**d**) (*n* = 5). **e** Microscopy images of GAS muscles immunostained with anti-MyHC antibodies (*n* = 5). **f** Quantification of SDH-positive fibers in GAS muscles via SDH staining (*n* = 5). *P < 0.05, **P < 0.01.

**Fig. 4:**
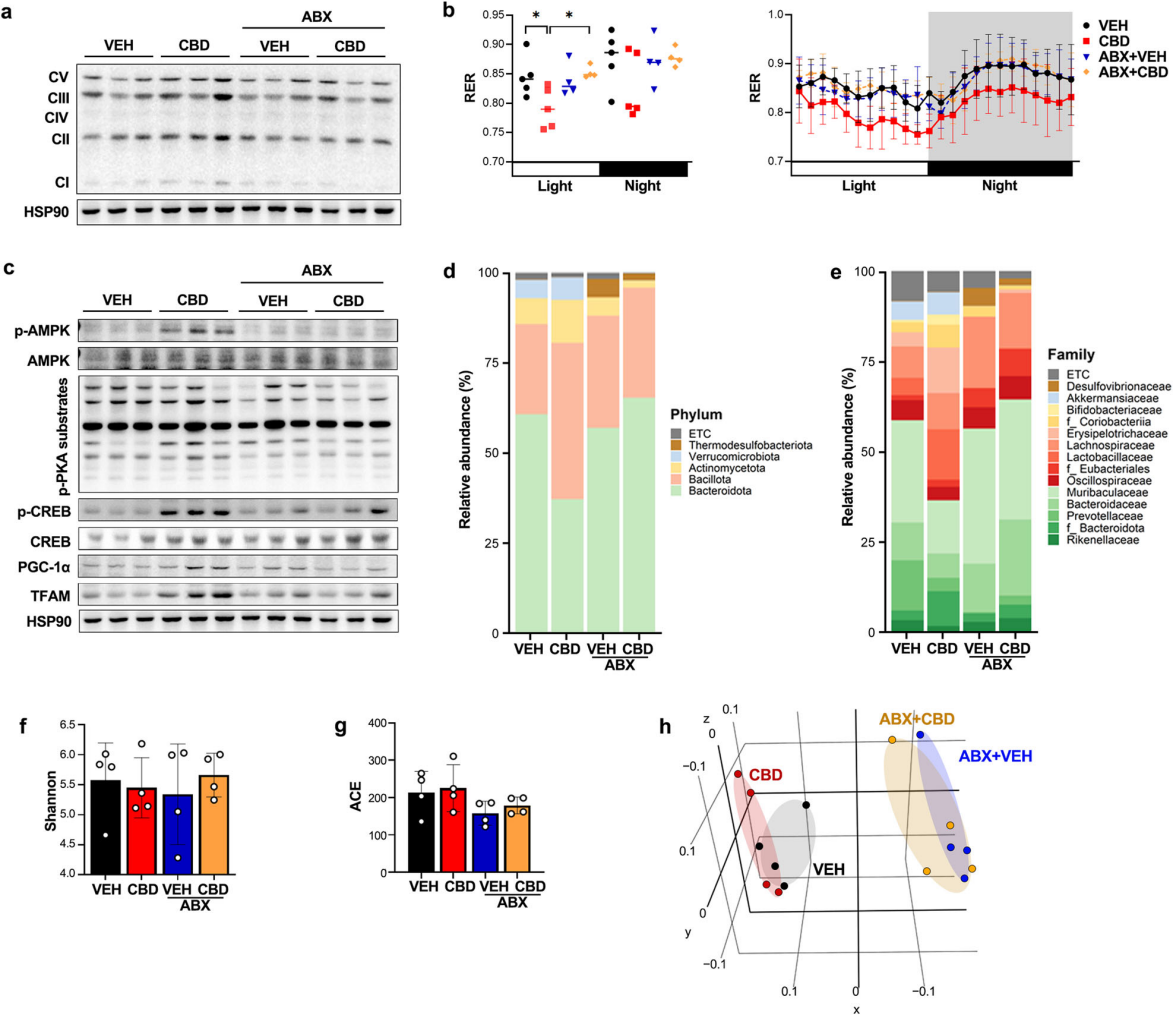
Antibiotic treatment attenuates the effect of CBD on skeletal muscle. **a** Western blot analysis of the expression of OxPhos subunits in GAS muscles (*n* = 5). **b** The 24-h RER was measured by indirect calorimetry (*n* = 5). **c** Western blot analysis of the degree of mitochondrial biogenesis in GAS muscles (*n* = 5). **d**, **e** The relative abundance of bacteria, as analyzed through 16S rDNA amplicon sequencing, at the phylum (**d**) and family (**e**) levels (*n* = 4). **f**, **g** The α-diversity of the VEH and CBD treatment groups was assessed via the Shannon (**f**) and ACE (**g**) indices (*n* = 4). **h** β-Diversity analysis was performed via unweighted UniFrac metrics (*n* = 4). *P < 0.05, **P < 0.01.

Doxycycline was administered on the same day as CBD, with a time interval between doses, and did not affect body weight or food intake in the mice (Fig. 3a and Supplementary Fig. 3a, b). ABX cotreatment blocked the CBD-induced improvements in running performance and oxidative muscle fiber density, as well as mitochondrial gene expression in GAS muscles (Figs. 3b–f and 4a and Supplementary Fig. 3c, d). Further analyses revealed that ABX prevented the CBD-driven reduction in the RER (VCO_2_/VO_2_) and the activation of AMPK, p-CREB and PGC-1α (Fig. 4b, c and Supplementary Fig. 3e). Microbiome analysis revealed that ABX offset the CBD-induced increases in Bacillota and Actinobacteria, suggesting that microbiome changes were crucial to the exercise performance effects of CBD (Fig. 4d). At the family level, the abundance of Erysipelotrichaceae and Bifidobacteriaceae, which also increased, was offset by antibiotic treatment (Fig. 4e). For α-diversity, there were no significant differences due to CBD or ABX treatment (Fig. 4f, g), but for β-diversity, the analysis of the unweighted UniFrac distance metric showed significant differences for each group (*P* = 0.002) because there were more changes in the proportion of species with low abundance than in the proportion of species with high abundance (Fig. 4h). These results suggest that the altered gut microbiome caused by CBD administration may have contributed significantly to the effects on exercise performance.

### *Bifidobacterium animalis* is significantly increased by CBD treatment, improves exercise performance and increases oxidative muscle fibers

**Fig. 5:**
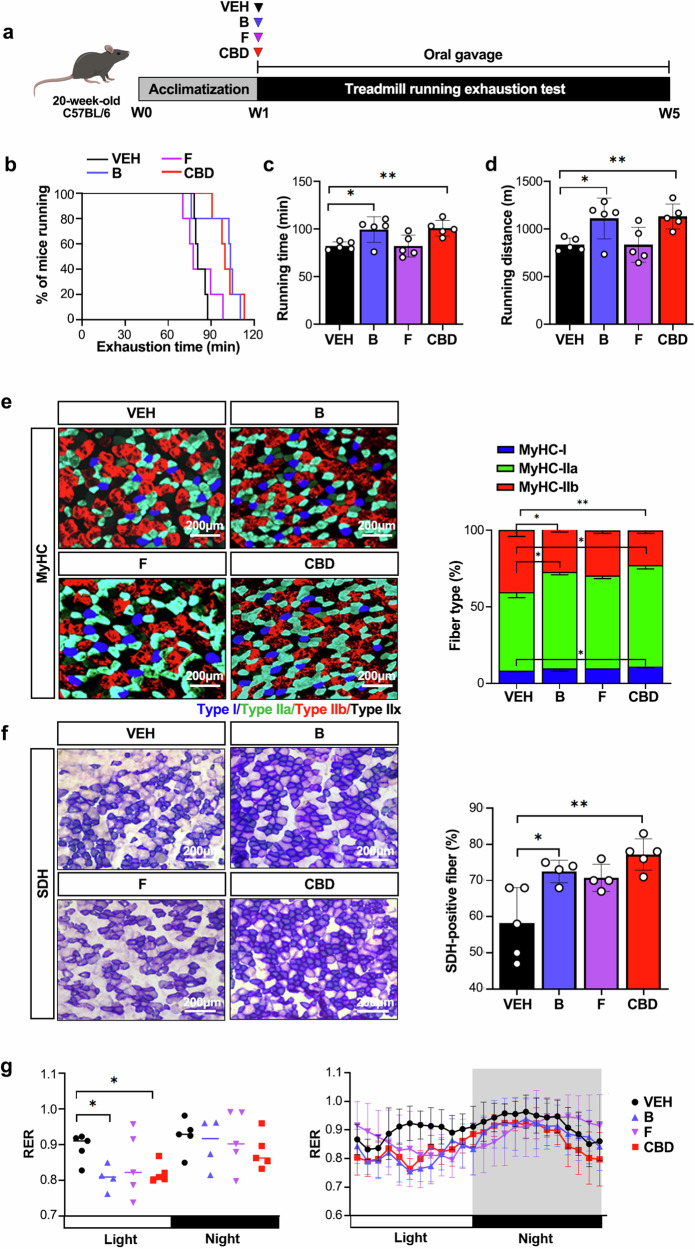
Treatment with *Bifidobacterium animalis* leads to improved exercise performance in mice. **a** The experimental design for assessing the effects of bacteria B (*Bifidobacterium animalis*) and bacteria F (*Faecalibaculum rodentium*) on exercise performance. **b** Running population plotted against time to exhaustion (*n* = 5). **c**, **d** Average running time (**c**) and distance until exhaustion (**d**) (*n* = 5). **e** Microscopy images of GAS muscles immunostained with anti-MyHC antibodies (*n* = 5). The results are plotted as a percentage of the fibers per muscle. **f** Quantification of SDH-positive fibers in GAS muscles via SDH staining (*n* = 5). **g** The 24-h RER was measured by indirect calorimetry. (*n* = 5). *P < 0.05, **P < 0.01.

**Fig. 6:**
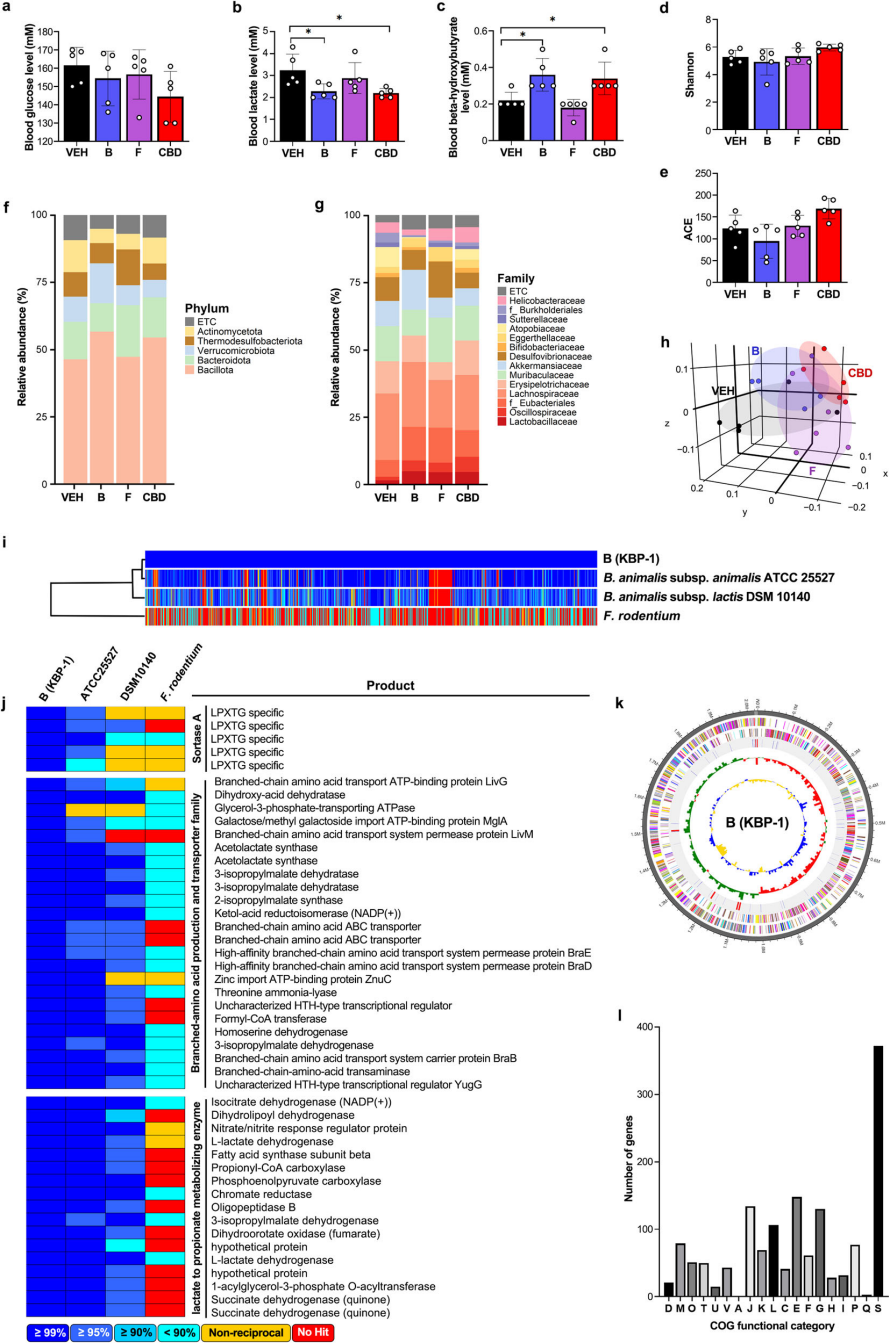
Treatment with *Bifidobacterium animalis* changes energy metabolism and the gut microbiome. **a**–**c** Blood glucose (**a**), lactate (**b**), and β-hydroxybutyrate (**c**) concentrations were measured (*n* = 5). **d**, **e** Effects of bacterial administration on the gut microbiota composition: α-diversity was analyzed through the Shannon (**d**) and ACE indices (**e**) in the VEH, bacteria B (*Bifidobacterium animalis*), bacteria F (*Faecalibaculum rodentium*) and CBD treatment groups, as determined by 16S rDNA amplicon sequencing (*n* = 5). **f**, **g** The relative abundance of bacteria at the phylum (**f**) and family (**g**) levels (*n* = 5). **h** β-Diversity analysis was performed with unweighted UniFrac metrics (*n* = 5). **i**, **j** Comparative analysis using publicly available databases and whole-genome sequences was performed via the UPGMA dendrogram method to construct a neighbor-joining tree based on the OrthoANI distance matrix and represented in Newick format (**i**). The pairwise ortholog matrix table was generated and visualized with color to reflect the degree of similarity between the corresponding sequences (**j**). **k** The pseudochromosome was drawn from 2 contigs. The outermost ring depicts the contigs. The next inner ring uses color codes to represent the CDS information analyzed on the forward strand, whereas the following inner ring displays the CDS information from the reverse strand. Moving inward, the fourth ring indicates tRNA (blue) and rRNA (red) locations. The innermost rings show the GC skew metrics (green for values higher than the average and red for values lower than the average) and the GC ratio metrics (blue for values higher than the average and yellow for lower values). Both the GC skew and the GC ratio metrics are presented at 10-kb intervals. **l** Analysis of COG functional categories and the number of genes within them for KBP-1: D, 19; M, 79; O, 51; T, 50; U, 15; V, 43; A, 1; J, 134; K, 69; L, 106; C, 41; E, 148; F, 61; G, 130; H, 28, I, 32, P, 77; Q, 3; and S, 372. The COG functional categories are described in Supplementary Table 2. *P < 0.05.

We next selectively isolated specific microbes whose abundance significantly increased following CBD administration. The microorganisms included *Faecalibaculum rodentium*, which belongs to the Erysipelotrichaceae family, and *Bifidobacterium animalis*, which belongs to the Bifidobacteriaceae family. The single-bacteria-treated groups were named after the initials of each strain; group B was treated with *Bifidobacterium animalis*, and group F was treated with *Faecalibaculum rodentium*. The selected strains are species whose abundance significantly increased according to microbiome data analysis (Supplementary Fig. 4). Each microbe was administered individually at equal concentrations, and the results were compared with the exercise performance-enhancing effects of CBD (Fig. 5a). The comparison revealed that endurance capacity, represented by average running time, running distance and running time to fatigue, increased significantly in the bacteria B- or CBD-treated groups but not in the bacteria F-treated group (Fig. 5b–d). Food intake, as well as body and muscle weights, did not differ between the groups (Supplementary Fig. 5a–d). The immunostaining and qPCR results for myofiber marker genes also revealed a shift toward an oxidative myofiber type and an increase in SDH-positive fiber density in the bacteria B treatment group, which was consistent with the effects of CBD (Fig. 5e, f and Supplementary Fig. 5e). Indirect calorimetric analysis revealed a decrease in the RER in bacteria B-treated mice, indicating improved fatty acid utilization, which is consistent with the effects of CBD (Fig. 5g). In addition, treatment with bacteria B decreased serum lactate and increased ketone body β-hydroxybutyrate levels, with no changes in blood glucose levels, indicating improved exercise endurance (Fig. 6a–c). We also measured the serum and fecal SCFA levels. While CBD increased the serum concentrations of acetic acid and propionic acid and bacteria B and F also increased the serum propionic acid concentration, the concentration of butyric acid did not significantly change across the treatment groups (Supplementary Fig. 5f). In feces, acetic acid and butyric acid levels were slightly decreased by bacteria F treatment, with no changes observed resulting from either CBD or bacteria B (Supplementary Fig. 5g, h). These results show that the bacteria B treatment improves exercise endurance through changes in muscle fiber type and metabolic substrate utilization.

### *Bifidobacterium animalis,* named KBP-1, alters energy metabolism and the gut microbiome

Interestingly, treatment with bacterium B decreased serum lactate levels and increased serum ketone levels, supporting its role in improving exercise endurance (Fig. 6a–c). Analysis of the gut microbiome composition revealed no effect on α-diversity but did reveal significant compositional differences, with principal coordinate analysis via unweighted UniFrac indicating a significant (*P* = 0.001) separation between groups (Fig. 6d–h). To further investigate mechanisms beyond microbiome changes that contribute to the muscular endurance-enhancing effects of bacterium B, we performed a whole-genome analysis of the strain. We identified and classified the bacterium as *Bifidobacterium animalis* KBP-1 and sought to characterize KBP-1 via publicly available databases. For comparison, we chose *B. animalis* subsp*. animailis* ATCC 25527 and *B. animalis* subsp. *lactis* DSM 10140, which were determined to be most similar to KBP-1, and *Faecalibaculum rodentium*, the bacteria F species that has not been shown to improve muscular endurance. The similarity between single strains was analyzed via a pairwise ortholog matrix, with darker blue indicating greater similarity and red indicating less similarity. Consistent with the similarity results, the phylogenomic analysis of the single strains also revealed separation (compared with KBP-1, 98.97%, 95.56% and 64.91% from top to bottom) (Fig. 6i). Pairwise ortholog matrix analysis revealed that KBP-1, which we isolated and used in our experiments, is characterized by high expression of sortase A, branched-chain amino acid (BCAA) production and secretion proteins, and lactate-metabolizing enzymes (Fig. 6j). Whole-genome analysis of KBP-1 revealed that the total genome size was 2,209,436 bp, the GC content was 60.4%, the CDS count was 1,640, the number of rRNA genes was 16, the number of tRNA genes was 54, the sequencing depth of coverage was 377.0× and the number of contigs was 2, which were analyzed according to chromosome type (Fig. 6k). Analysis of the Clusters of Orthologous Groups (COGs) functional categories for KBP-1 and the associated gene count revealed that the amino acid transport and metabolism genes, classified under category E, are linked to the production and transport of BCAAs, which are highly expressed in KBP-1 (Fig. 6l). These findings suggest the strong involvement of these genes in enhancing muscular endurance.

## Discussion

In this study, we identified a novel function of CBD in reprogramming myofibers to an oxidative type and enhancing mitochondrial biogenesis, thereby improving muscular endurance. CBD activates the AMPK and PKA–CREB pathways, both of which are linked to PGC-1α activation^36,49^, which regulates mitochondrial dynamics and skeletal muscle function^50^. AMPK directly phosphorylates PGC-1α, thereby regulating mitochondrial biogenesis and fatty acid oxidation in skeletal muscle^51^. Mechanistically, we observed significant changes in the gut microbiome in CBD-treated animals, and surprisingly, both the mitochondrial biogenesis and exercise performance-enhancing effects of CBD were abolished by antibiotic administration. Finally, we isolated a strain, named KBP-1, from CBD-treated mice that exhibited endurance-enhancing effects by altering myofiber composition.

The hypothesis that changes in the gut microbiome in response to CBD administration may affect exercise performance was supported by the antibiotic cotreatment findings. To conduct this experiment, we carefully selected ABX, as even a single microbial species can be critical. Some ABX can cause weight loss and digestive disorders, such as inflammatory bowel disease and anxiety/depressive behavior^52,53^, and it has been reported that ABX-treated mice exhibit reduced exercise performance and muscular endurance^48,54^, while colonizing germ-free mice with *Bacteroides fragilis* restores endurance^54^. To achieve this goal, we administered various ABX to compare changes in the microbiome of mice and selected doxycycline, as it exhibited bactericidal effects on Erysipelotrichaceae and Bifidobacteriaceae, which were significantly increased by CBD administration but showed no significant change overall (Supplementary Fig. 2). Furthermore, doxycycline did not cause changes in body weight, a common side effect of antibiotic treatment (Supplementary Fig. 3a). Our study in which doxycycline was coadministered with CBD revealed that the effects of CBD on muscle exercise capacity, myofiber reprogramming, mitochondrial biogenesis and the RER were abolished. These results suggest that the athletic performance-enhancing effects of CBD are primarily due to the gut microbiome.

A systematic review revealed that the gut microbiome and skeletal muscle function are closely related, with potential mechanisms associated with energy metabolism, inflammation and mitochondrial function^55^. We were able to isolate two strains from the gut microbiome whose abundance significantly increased with CBD administration: *Bifidobacterium animalis*, which belongs to the Bifidobacteriaceae family, named B or KBP-1, and *Faecalibaculum rodentium*, which belongs to the Erysipelotrichaceae family, named F. Although the abundance of *Allobaculum stercoricanis* also significantly increased (Supplementary Fig. 4), identification revealed that this is a new strain, not a known one. We did not include it in this study, but we would like to explore this strain in future experiments. We observed increased muscular endurance in bacteria B (KBP-1)-treated mice and performed whole-genome sequencing to understand the underlying mechanism involved. To further analyze how KBP-1 differs from other *B. animalis* or *F. rodentium* strains, we compared it with *B. animalis* subspecies *animalis* ATCC 25527, *B. animalis* subsp. *lactis* DSM 10140 and *F. rodentium* Alo17 whole-genome sequences in publicly available databases. The results showed that KBP-1 is characterized by high expression of biosynthetic enzymes for BCAAs and their export pump. BCAAs positively impact fatigue, muscle damage, exercise capacity and lipid oxidation during endurance exercise after muscle glycogen depletion, making them useful supplements for promoting muscular endurance^56–58^. Future studies exploring the effects of CBD or KBP-1 on BCAA production would enhance our understanding. We also found that the levels of enzymes involved in lactic acid metabolism, such as lactate dehydrogenase, pyruvate carboxylase, pyruvate:ferredoxin oxidoreductase, SDH, propionyl-CoA carboxylase and dihydrolipoamide dehydrogenase, were increased by KBP-1 (Fig. 6j–l), which could contribute to reduced blood lactic acid levels in CBD-treated mice. In addition, the ketone body β-hydroxybutyrate, which is increased by KBP-1 and CBD, may contribute to improved endurance, as ketone bodies serve as an excellent alternative fuel source for peripheral tissues, including the heart and skeletal muscle^59^. During exercise, ketone bodies are oxidized as fuel, and their metabolic actions can reduce proteolysis in skeletal muscle^59^.

Other microbial metabolites may also have contributed to the improved exercise performance. SCFAs, particularly acetic, propionic and butyric acids, are key gut microbiome metabolites linked to athletic performance, supporting energy production, gut health, anti-inflammatory effects and various physiological processes^60^. However, this study did not observe a marked increase in serum SCFAs that would support enhanced muscular endurance by KBP-1 or CBD, except for serum propionic acid, which was slightly and similarly elevated by both KBP-1 and CBD. Furthermore, we observed a discrepancy between the serum and fecal SCFA levels, which may be attributed to differences in the freshness of the serum and feces; we measured SCFAs in fresh serum versus older feces. In addition, these levels could be influenced by various factors, such as metabolism by the liver, epithelial cells of the gastrointestinal tract or even the gut microbiome itself. Taken together, our results suggest that SCFAs are not the primary contributors to endurance enhancement through these treatments.

We cannot exclude the possibility that CBD may exert a direct effect on skeletal muscle, enhancing exercise performance, while the gut microbiome plays a primary role. The presence of endogenous cannabinoid receptors, such as CB1 and CB2, and other mediators involved in relaying the cellular functions of CBD has been reported. The potential direct action of CBD on skeletal muscle is supported by the following findings: first, skeletal muscles express cannabinoid receptors^61,62^; second, our in vitro treatment of C2C12 cells with 20 μM CBD increased the oxygen consumption rate (Supplementary Fig. 1g); third, the extent to which *B. animalis* KBP-1 improved exercise performance was less than that of CBD treatment.

In summary, we propose that both CBD and the gut bacteria *B. animalis* KBP-1, which is increased by CBD treatment, could be used in strategies to promote endurance exercise performance. Nevertheless, we recognize certain limitations of this study. First, the limited sample size reduces the generalizability and statistical power of the study. Second, although we suggest that KBP-1 enhances BCAA production, we did not measure BCAA levels in serum or muscle tissue. These limitations could be addressed in future studies with larger sample sizes, focusing on reproducibility under various conditions and further investigating the specific efficacy of KBP-1.

## Supplementary information

Supplementary Information
