# Supplementary material for: Cannabidiol reshapes the gut microbiome to promote endurance exercise in mice
Source: Exp Mol Med. 2025 Feb 18;57(2):489–500. doi: 10.1038/s12276-025-01404-5 (PMC11873264; doi:10.1038/s12276-025-01404-5)
Supplement: Supplementary file 1 — Supplementary Information [file 12276_2025_1404_MOESM1_ESM.pdf]

[SUPPLEMENT]

Cannabidiol reshapes the gut microbiome to promote endurance exercise in mice

Supplementary Table 1. Sequences of primers for qPCR

Gene	Sequences for primers (5'-3')
Myh7	FOR: ACAAGCTGCAGCTGAAGGTG
	REV: TCATTCAGGCCCTTGGCAC
Myh2	FOR: CCAGCTGCACCTTCTCGTTTGCCAG
	REV: CATGGGGAAGATCTGGTCTTCTT
Myh4	FOR: CCTGGAACAGACAGAGAGGAGCAGGAGAG
	REV: GTGAGTTCCTTCACTCTGCGCTCGTGC
Myh1	FOR: TGCAACAGTTCTTCAACCAC
	REV: GCCAGGTCCATCCCAAAGT
Tnni1	FOR: TGAAGCCAAATGCCTCCACAACAC
	REV: ACACCTTGTGCTTAGAGCCCAGTA
Tnnc1	FOR: AGCTCATGAAGGACGGTGACAAGA
	REV: AACCGTGCAAGACCAGCATCTACT
Tnnt1	FOR: AAGGGGAGCGTGTGGATTTG
	REV: TCCTCCTTTTTCCGCTGTTCA
Tnnt3	FOR: AACTGGAGACTGACAAATTCGAGT
	REV: GCTGTGCTTCTGGGTTTGGT
Tnnc2	FOR: CCATCATCGAGGAGGTGGAC
	REV: CTTCCCCTTCGCATCCTCTT
Tnni2	FOR: GCACCTGAAGAGTGTGATGCT
	REV: TCTCCTTCTCAGATTCTCGGC
Mb	FOR: CATGGTTGCACCGTGCTCACAG
	REV: GAGCCCATGGCTCAGCCCTG
Sdhb	FOR: CAGAGTCGGCCTGCAGTTTC
	REV: GGTCCCATCGGTAAATGGCA
Fndc5	FOR: TCCTCTTCATGTGGGCAGGT
	REV: GGGCTCGTTGTCCTTGATGATA
Nrf1	FOR: GGAGCACTTACTGGAGTCC
	REV: CTGTCCGATATCCTGGTGGT
Tfam	FOR: GCAAAGGATGATTCGGCTCAGGGAA
	REV: CCGGATCGTTTCACACTTCGACGG
Mtco1	FOR: CTACTATTCGGAGCCTGAGC
	REV: GCATGGGCAGTTACGATAAC
Mtco2	FOR: AACCATAGGGCACCAATGATAC
	REV: GGATGGCATCAGTTTTAAGTCC
Mcad	FOR: GGTTTGGCTTTTGGACAATG
	REV: TGACGTGTCCAATCTACCACA
Atp5o	FOR: TCTCGACAGGTTCGGAGCTT
	REV: AGAGTACAGGGCGGTTGCATA
Cox5b	FOR: TTCAAGGTTACTTCGCGGAGT
	REV: CGGGACTAGATTAGGGTCTTCC
Cycs	FOR: CCAAATCTCCACGGTCTGTTC
	REV: ATCAGGGTATCCTCTCCCCAG
Ndufs1	FOR: TGCAAATCCCTCGATTCTGTTAC
	REV: GCTTTCTCAATCTCTACCAGGC
Ndufv2	FOR: GCAAGGAATTTGCATAAGACAGC
	REV: TAGCCATCCATTCTGCCTTTG

Gapdh	FOR:	GGCATGGACTGTGGTCATGA
	REV:	TTCACCACCATGGAGAAGGC
Ppia	FOR:	GCATACGGGTCCTGGCATCTTGTCC
	REV:	ATGGTGATCTTCTTGCTGGTCTTGC

forward, FOR; reverse, REV

Supplementary Table 2. COG functional categories description

COG categories	Functional descriptions
D	cell cycle control, cell division, chromosome partitioning
M	cell wall, membrane, envelope biogenesis
O	post-translational modification, protein turnover, and chaperones
T	signal transduction mechanisms
U	intracellular trafficking, secretion, and vesicular transport
V	defense mechanism
A	RNA processing and modification
J	translation, ribosomal structure, and biogenesis
K	transcription
L	replication, recombination, and repair
C	energy production and conversion
E	amino acid transport and metabolism
F	nucleotide transport and metabolism
G	carbohydrate transport and metabolism
H	coenzyme transport and metabolism
I	lipid transport and metabolism
P	inorganic ion transport and metabolism
Q	secondary metabolites biosynthesis, transport, and catabolism
S	function unknown

Supplementary Figure

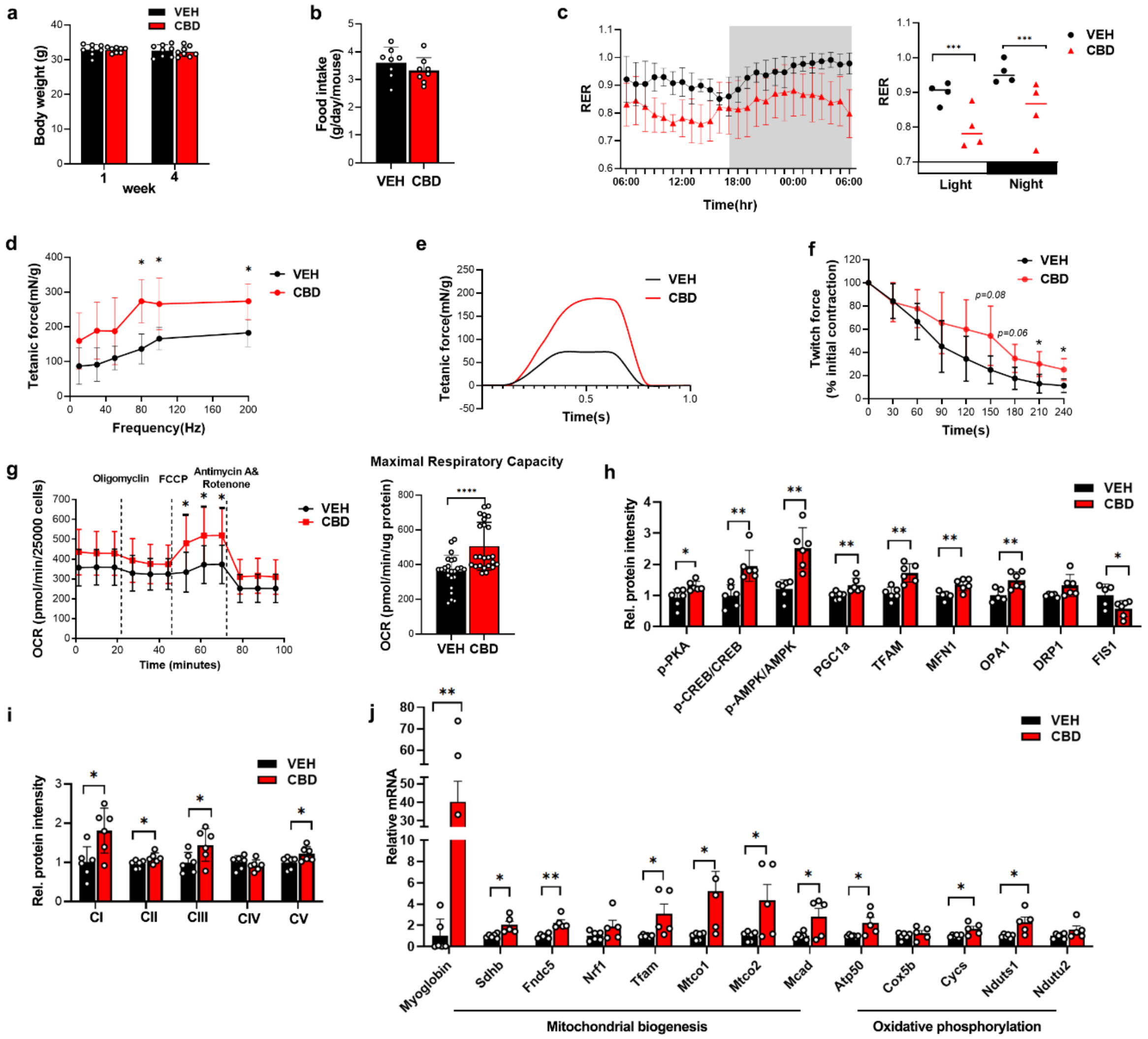

Supplementary Figure 1. CBD treatment causes in vivo and in vitro improvements in muscle endurance

(a, b) Body weight change after the 1- and 4-week drug treatment and exercise training in mice, and their daily food intake (n = 8). (c) The twenty-four-hour light and dark cycle respiratory exchange ratio (RER) was measured by indirect calorimetry in the mice used for Fig. 1 (n = 4). (d, e) A separate cohort of mice was administered CBD (30 mg/kg) or vehicle for 4 weeks without exercise training, and skeletal muscle function was measured by electrical pulse stimulation (EPS). Tetanic force-frequency relationships were determined by triggering contraction using incremental stimulation frequencies (1 ms pulses at 10-200 Hz for 500 ms at 100 V, n = 4). (f) The fatigue index was measured at 1 Hz and 100 V by repeated stimuli for 4 min and expressed as a percentage of the initial contractile force (n = 4). (g) Oxygen consumption rate (OCR) was measured using a Seahorse XF analyzer in differentiated C2C12 myotubes. Maximal respiration (difference between OCR after FCCP and antimycin A

(AA)/rotenone injection) was determined after incubation with CBD or vehicle for 24 h (n = 8). (h) The relative protein intensity analysis of the expression of mitochondrial biogenesis genes (n = 6) and (i) OxPhos subunits in gastrocnemius (GAS) muscles (n = 6) via western blotting. (j) qPCR analysis of mitochondrial biogenesis and oxidative phosphorylation (n = 5).

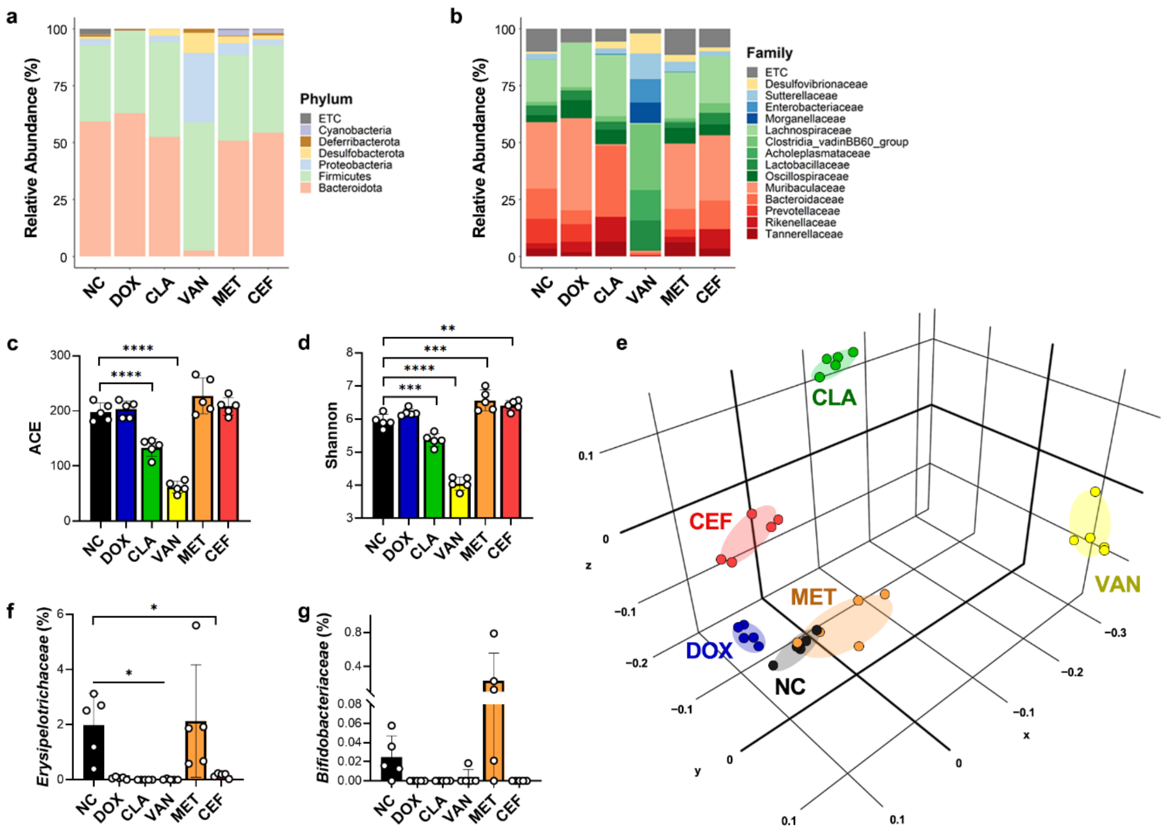

Supplementary Figure 2. Different classes of antibiotics cause different gut microbiome changes

For comparison by antibiotic class, doxycycline (DOX), clarithromycin (CLA), vancomycin (VAN), metronidazole (MET), and cefaclor (CEF) were used to compare changes in microbial composition. (a, b) The impact of antibiotic administration on the relative abundance of bacteria at the phylum and family level (n = 5). (c, d) The α-diversity is analyzed using the Ace and Shannon index (n = 5). (e) The β-diversity analysis used unweighted UniFrac metrics (n = 5). (f, g) The ratio of *Erysipelotrichaceae* and *Bifidobacteriaceae* at the family level was compared in the NC and antibiotic-treated groups (n = 5).

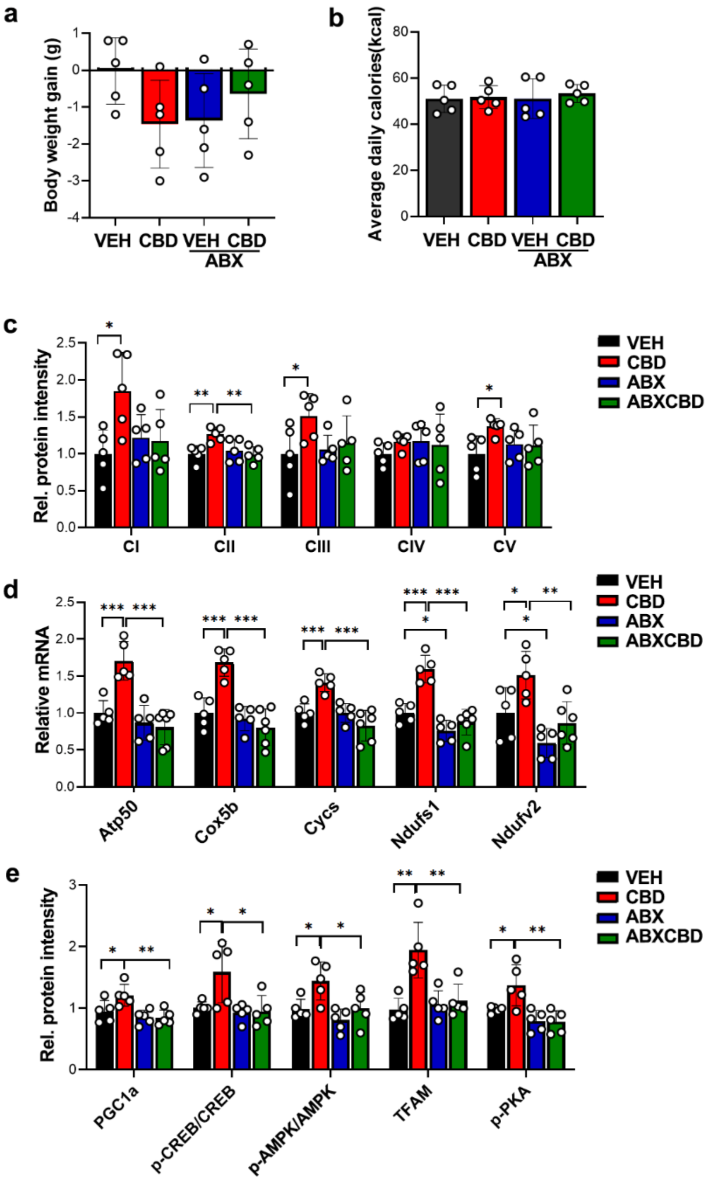

Supplementary Figure 3. Antibiotic treatment negates CBD-induced muscle function changes

(a-b) Body weight change after the 4-week exercise and daily food intake of mice (n = 5). (c) Quantification results of western blot analysis of OxPhos complex (n = 5). (d) qPCR analysis of genes related to oxidative phosphorylation (OxPhos) in GAS (n = 5). (e) Quantification results of western blotting analysis of the genes involved in mitochondrial biogenesis in GAS (n = 5).

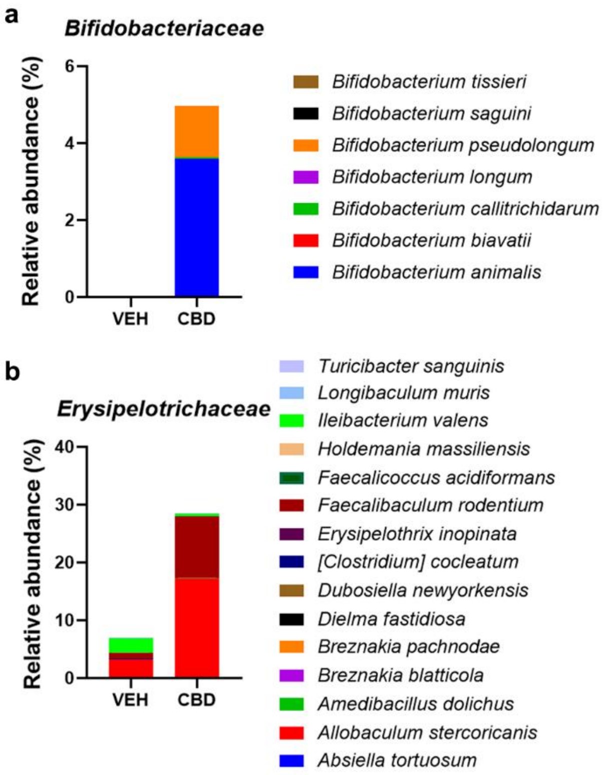

Supplementary Figure 4. The rationale for single bacteria selection for administration

Comparison of the relative abundance of species belonging to the (a) *Bifidobacteriaceae* and (b) *Erysipelotrichaceae* family between the VEH and CBD groups (n=5).

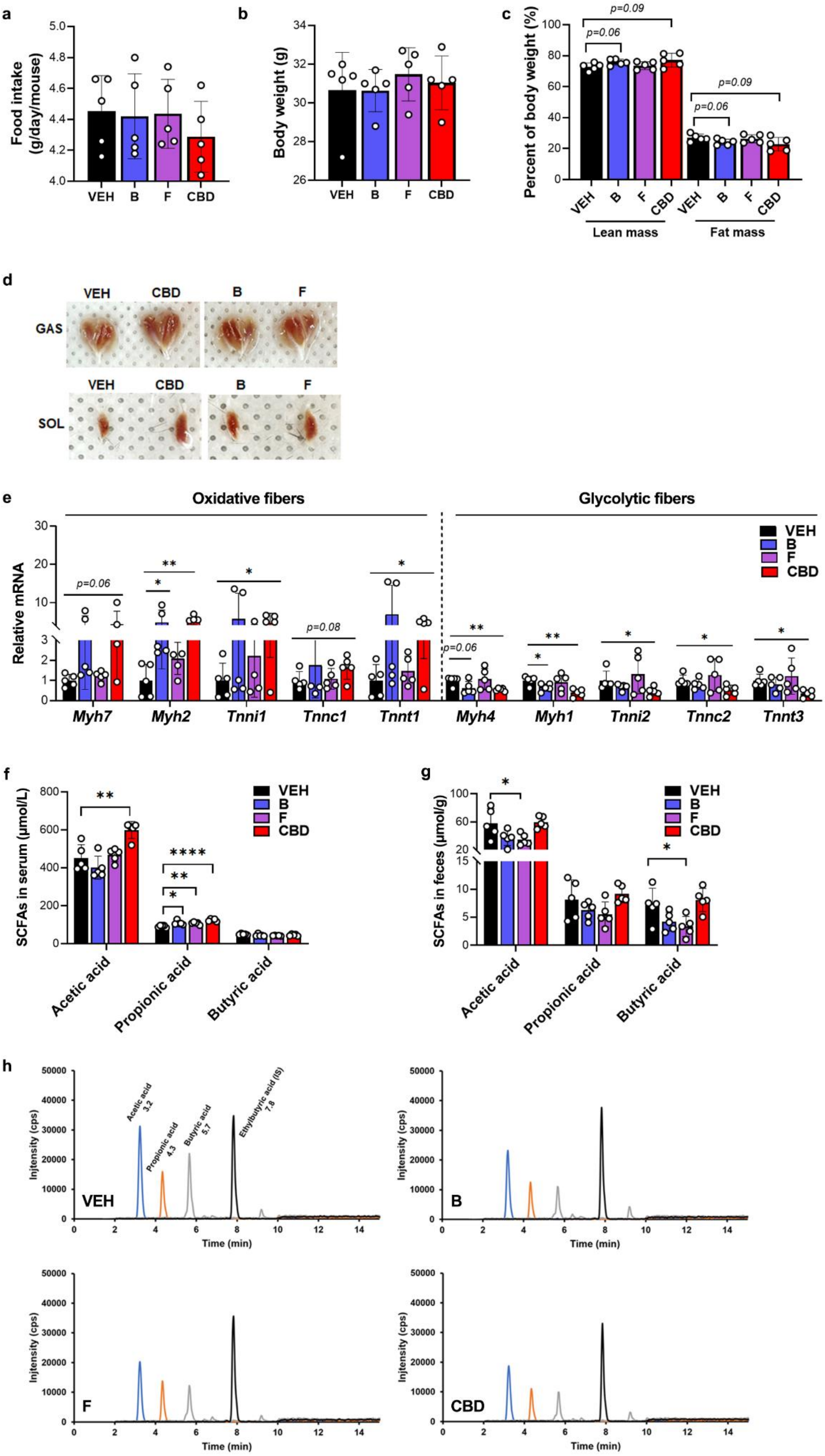

a
Food intake (g/day/mouse)
VEH
B
F
CBD
b
Body weight (g)
c
Percent of body weight (%)
p=0.09
p=0.06
Lean mass
Fat mass
d
GAS
SOL
e
Oxidative fibers
Glycolytic fibers
Relative mRNA
p=0.06
p=0.08
Myh7
Myh2
Tnni1
Tnnc1
Tnnt1
Myh4
Myh1
Tnni2
Tnnc2
Tnnt3
f
SCFAs in serum (μmol/L)
Acetic acid
Propionic acid
Butyric acid
g
SCFAs in feces (μmol/g)
h
Injtensity (cps)
Time (min)
Acetic acid 3.2
Propionic acid 4.3
Butyric acid 5.7
Ethylbutyric acid (IS) 7.8

Supplementary Figure 5. Administration of Bacteria B produces similar metabolic activity changes and muscle endurance improvements as CBD

(a-c) Body weight, fat and lean mass after the 4-week exercise, daily food intake of mice (n = 5). (d) Representative photographs show skeletal muscles from the vehicle and CBD treatment mice. (e) mRNA levels of known markers of oxidative and glycolytic fibers were analyzed by qPCR. mRNA levels were standardized against Gapdh and plotted relative to the expression in vehicle-treated mice (n = 5). (f) Short-chain fatty acid in serum (n = 5), (g) fecal (n = 5) by LC-MS/MS, and (h) representative LC-MS/MS chromatograms of monomers measured in feces are expressed per group.
